# The impact of infectious disease consult on a hospitalist prescribing metric of broad-spectrum antibiotics

**DOI:** 10.1017/ash.2025.10190

**Published:** 2025-10-14

**Authors:** Lucy S. Witt, Radhika Prakash Asrani, Hyun Bin Kim, Chad Robichaux, Jessica R. Howard-Anderson, Scott K. Fridkin

**Affiliations:** 1 Division of Infectious Diseases, Emory University School of Medicine, Atlanta, GA, USA; 2 Georgia Prevention Epicenters, Atlanta, GA, USA; 3 Department of Medicine, Emory University School of Medicine, Atlanta, GA, USA; 4 Department of Biomedical Informatics, Emory University School of Medicine, Atlanta, GA, USA

## Abstract

We explored the impact of infectious disease (ID) consultations on hospitalists’ prescribing of broad-spectrum, hospital-onset (BSHO) antibiotics. Periods with more ID consults had increased BSHO-DOT; however, this relationship was nonlinear, and ID consult frequency did not explain variability in prescribing. ID consultation should be considered when creating prescriber performance metrics.

## Introduction

Inappropriate antimicrobial use causes harms to patients and the healthcare system.^1^ The Centers for Medicare and Medicaid Services requires hospitals to develop and implement antimicrobial stewardship programs, and antimicrobial use is routinely being reported to the National Health Safety Network (NHSN).^2,3^ There are many activities aimed at improving antimicrobial use including education, restrictions, peer-to-peer comparison, audit-and-feedback, and electronic medical record (EMR)-based nudges. Although all methods may impact change, peer-to-peer comparison and audit and feedback strategies have been shown to be effective in improving antimicrobial use in randomized and non-randomized trials. ^4,5^

As part of an ongoing trial, hospitalists within our healthcare system receive a peer-to-peer comparative prescribing metric: an observed-to-expected ratio (OER) of days of therapy (DOT) for NHSN-defined broad-spectrum, hospital-onset (BSHO) antibiotics.^6^ Prior studies have shown that infectious disease (ID) consultants affect antibiotic use, usually with improvements in appropriateness and decreased antibiotic use overall.^7^ Here we quantify the impact of ID consultations on hospitalists’ OER in the pretrial period to build credibility for this prescribing metric.

## Methods

Provider-specific prescribing metrics were generated from antibiotic administration data from the EMR and attributed to a hospitalist based on billing data. The methods for generating the risk-adjusted OER have been previously described; provider specific OERs are a standardized metric based on observed and estimated DOT, the latter calculated using linear models incorporating billed patient days and the proportion of each providers’ patients with key predictors of DOT including sepsis, urinary tract infection, and end-stage renal disease (determined by ICD-10 code) in each bimonthly period.^8^ The OER is calculated every 2 months for all hospital medicine providers at 5 hospitals within a single healthcare system. All hospitals have antimicrobial stewardship programs and have ID consultation provided by private groups (community hospitals) or university-affiliated groups(academic hospitals). The encounter was considered to have an ID consult if an ID provider billed at any time during the encounter. The ID consult indicator was validated through chart review in a random subset of 25 patients cared for by 52 providers. This validated that (1) all patients with an ID consult indicator received a consultation from an ID provider, (2) in 85% of the encounters the hospitalist’s prescribing of BSHO antibiotics was within a 3-day window of the ID consultant’s involvement, and (3) in 60% of encounters, the ID consultant recommended maintaining or escalating to BSHO antibiotics. For each provider, we calculated the percentage of encounters with ID consultation (consult density). We then stratified the providers’ consult density into quintiles equating to the approximate ratio of encounters with an ID consultation (eg, 1 in 3, 1 in 4 patients). We assessed the effect of ID consult density on hospitalist-specific DOT using a linear mixed effects model with random intercepts for both provider and facility (nested) and adjusted for patient characteristics described above. BSHO-DOT for each stratum of ID consult density was calculated and compared. Distribution of OERs was compared before and after adjusting for ID consult density. Based on prior data and the distribution of OERs in our study, an OER > 1.25 was classified as “high.” Data analysis was performed in SAS 9.4 (Cary, NC). This project was approved by the Emory University IRB.

## Results

**Figure 1.**
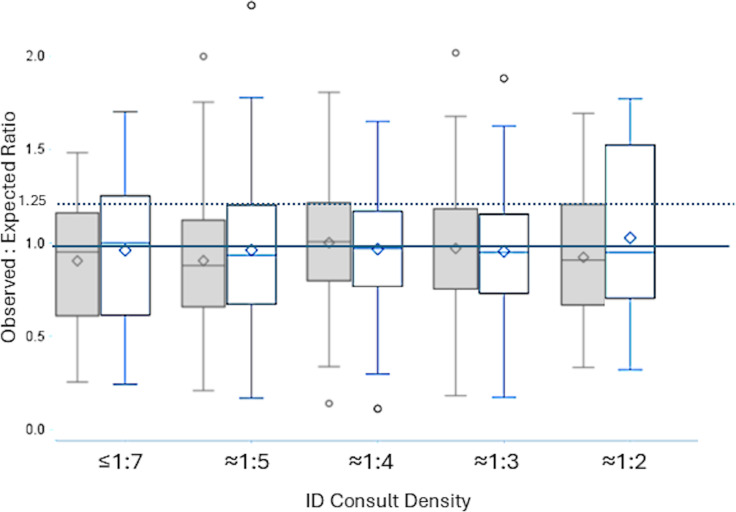
Distribution of observed-to-expected ratios (OERs) for providers, by rough proportion of patients having infectious disease (ID) consultation (ID consult density), both before (shaded) and after (white) adjusting for impact of ID consultation in the predictive models. Dashed line is the OER cutoff for high prescribing; solid line is an OER of 1.0, where observed days of therapy (DOT) = predicted DOT.

Over the 6-month period (January–June 2023), 154 unique providers cared for 53,815 patients, allowing for 458 bimonthly provider-periods, each producing a unique OER. Overall, 21% of these patients were evaluated by an ID consultant (facility-specific median range:19%–26%). Approximately two-thirds of provider-periods had an ID consult density of 15%–25%.The mean DOT observed increased as the ID consult density increased (Table 1). After adjusting for patient characteristics in multivariate models, we found that compared to hospitalists with an ID consult density of 1:7, the estimated DOT were significantly higher for hospitalists with an ID consult density of 1:3 (mean additional 3.4 DOT, 95% confidence interval [CI] 0.9–5.9) or 1:4 (mean additional 2.7 DOT, 95% CI 0.4–5.0). This effect was not significant in other strata and was not linear. The multivariate OER model fit was not greatly improved with the addition of an ID consult variable, and interfacility variability was observed (interclass correlation coefficient of 56%). The distribution of OERs changed slightly with the addition of the ID consult density variable (Figure 1). For the 82 provider-periods that were initially classified as “high” prescribers (OER > 1.25), 18 (22%) were no longer classified as high when the ID consult density variable was added to the model. Conversely, 15 of 346 (4%) of provider-periods with OER ≤ 1.25 moved into the “high” category with the inclusion of the ID consult variable.

**Table 1.** Days of therapy of broad-spectrum hospital-onset antibiotics by density of infectious disease consults

ID consult density		Provider-periods, no.	DOT observed, mean	DOT above referent	Adjusted estimate of DOT above referent			
Ratio ID consults to patients billed	Range in density				DOT	95% CI		*P*-value
1:2	34%–50%	11	11.5	2.4	.71	−3.52	4.94	.741
1:3	26%–33%	101	13.8	4.7	3.39	.9	5.88	.008
1:4	21%–25%	153	12.9	3.8	2.68	.37	5.00	.023
1:5	15%–20%	119	10.7	1.6	.75	−1.55	3.05	.523
1:7 (Ref)	0%–14%	44	9.1	Ref				

Note. DOT, days of therapy; CI, confidence interval; ID, infectious diseases.

Adjusted estimates account for the percentage of patients with ICD-10 codes for sepsis, urinary tract infection, and end-stage renal disease.

## Discussion

The duration of broad-spectrum antibiotics prescribed by hospitalists within our healthcare system is affected by ID consultations. Generally, a higher proportion of encounters with ID consultation led to an increase in DOT, but this was not noted for hospitalists who involved ID consultants in approximately half of patients, potentially due to the small sample size. This may also explain the effect of ID consultation being nonlinear. The impact of this effect on the risk-adjusted peer-comparative prescribing OER was modest, with a small reduction in variance. There was a small shifting of prescribing categories; approximately 20% of high prescriber-periods moved to non-high (OER < 1.25) when adding the ID consult variable. ID consult density does not account for most of the high prescriber-periods.

This sub-analysis was motivated by hospitalist feedback at our community hospitals, who commented that their prescribing habits for BSHO antibiotics were largely driven by ID consultants. Prior surveys have examined clinicians’ beliefs regarding the effect of ID consultation on their prescribing habits.^7^ Although a majority of those surveyed found ID input helpful, compliance with recommendations ranged from 35% to > 90% suggesting inconsistent uptake of ID recommendations.^7^ These findings are consistent with our data and suggest that the ID consultant’s effect on antibiotic prescribing is variable. Notably, prior studies in the United States have not examined the effect of ID consult on BSHO-DOT, which is an important metric currently being reported by acute care hospitals to NHSN and may soon be tied to reimbursement. Our study also found variation in the effect of ID consult density across our 5 hospitals, which likely speaks to differing relationships between ID consultants and hospitalists within our system.

Our study has many limitations. Although we validated our ID consult variable, for 15% of encounters with an ID consult the consultant’s note was not written within 3 days of BSHO prescribing by the hospitalist, making the consultant’s effect on this activity less clear. ID consult strata definitions were based on the general distribution of consult frequency but may have artificially separated providers with similar prescribing habits. Furthermore, a small percentage of provider-periods had an ID consult density of 34%–50%, making inferences about this group less precise. Lastly, we did not systematically evaluate the appropriateness of the prescribing activity, limiting the interpretation of our relationships to magnitude rather than quality of prescribing.

Our experience suggests that ID consultation has variable impact on hospitalist prescribed BSHO-DOT and does not fully explain high use. This influence should be considered when building credibility and reliability of prescribing performance; hospital specific culture should be examined to address perceived drivers of excessive prescribing even if the impact of the drivers are modest.
